# Cerebro-Spinal Meningitis

**Published:** 1864-07

**Authors:** 

**Affiliations:** Morgan Co., Ill.


ARTICLE XXVII.

### CEREBRO-SPINAL MENINGITIS.

BY DR. McVEY, of Morgan Co. Ill.

Presented to the Illinois State Medical Society, May, 1864

MR. PRESIDENT AND GENTLEMEN:

The subject of cerebro-spinal meningitis has engaged the attention of the profession for the last fifteen months. Certain portions of our country have suffered from an epidemic bearing the name referred to above. However, I think the name not very appropriate, as some of the patients die before inflammation could take place. Inflammation may follow as a secondary symptom. But the most prominent features of the disease in question, at the commencement, are those of congestion and depression, indicating the presence of some toxic agent in the circulating fluid, and that agent seems to have a very strong affinity for the brain, destroying the vitality of the blood, and overcoming the contractile power of the capillaries and vessels of the brain. Consequently, the circulation in brain is interrupted; the capillaries and the coats of the vessels in the brain expand to accommodate the accummulation of fluid in their structure, which is evidenced by the epistaxis that follow in some of the cases that recover. And, when reaction does take place, there is intense febrile excitement, with great restlessness and moaning. The eyes are suffused and the pupils are dilated. The patient is troubled with delirium, but, when aroused, for the most part answers questions correctly. Sometimes there are tremors of the whole body, one passing in succession after another. Sometimes there is squinting of one eye. The countenance has a brown hue. The tongue is slighted coated at the start, but, as the disease progresses, becomes thicker. The bowels are constipated. The patient vomits a dark grumous substance. The posterior cervical muscles contract and draw the head backwards. Sometimes there are petechia over the neck, breast, and extremities.

I have seen patients die within six hours after they were taken with coma and prostration, the circulation giving out first, intellection next, and lastly, respiration, without any febrile reaction whatever; but, in the majority of the cases, reaction does take place in about six hours after the attack.

In regard to treatment, the most successful I have met with has been cerebral and arterial stimulants, with arsenic as an anti-periodic. It is of the utmost importance to arouse the capillaries and vessels of the brain, in order that they may relieve themselves of the fluid accumulated in their structure, and I know of no better agent than opium to do it with. Under the foregoing treatment, I have had six patients recover, and three died. My partner, Dr. Brown, says he has treated, in the last six months, twenty-one cases, five of which were fatal, and sixteen recovered. If the symptoms are severe, viz.: Coma, muscular contraction, with pain in the limbs, back, and head, I have given opium in large doses, say to an adult, from 4 to 5 grains; arsenic freely, usually Fowler's solution, from 6 to 8 grs. every four hours; and where the muscular prostration continued for a considerable length of time, strychnia; alteratives moderately, keeping the bowels open with mild aperients, invariably applying friction to the surface; tincture capsicum and alcohol freely, with turpentine to the spine; sometimes blisters to the neck.

Convalescence is generally slow. Quinia, I think, contra-indicated, unless it is in very small doses, for the reason that it increases the agitation, and produces a certain amount of deafness, with high delirium. Probably, after the violence of the disease has passed off, it may be given with impunity, but not until the tremors have subsided, and the brain is so relieved that there is no danger of producing congestion of the eyes and ears; after which time it may have a salutary effect upon the digestive organs, and by giving tonicity to the system in general.

